# Separated hands further response–response binding effects

**DOI:** 10.3758/s13423-023-02419-7

**Published:** 2024-03-04

**Authors:** Silvia Selimi, Christian Frings, Birte Moeller

**Affiliations:** Cognitive Psychology, University of Trier, D-54286 Trier, Germany

**Keywords:** Action control, Binding and retrieval, Response-response binding, Response separation

## Abstract

Action control is hierarchically organized. Multiple consecutive responses can be integrated into an event representation of higher order and can retrieve each other upon repetition, resulting in so-called response–response binding effects. Previous research indicates that the spatial separation of responses can affect how easily they can be cognitively separated. In this study, we introduced a barrier between the responding hands to investigate whether the spatial separation of two responses also influences response–response binding effects. In line with previous research on stimulus–response binding, we expected an increased separability of responses to result in stronger response–response binding effects when responding hands were separated by a barrier. We indeed found stronger response–response binding effects with separated hands. Results indicate that a more distinct representation of individual actions through increased separability might benefit the control of hierarchical actions.

## Introduction

Everyday actions can be as simple as pressing a button and as complex as preparing a burger menu ordered in a fast-food restaurant. Yet, it is widely assumed that action control is hierarchically organized (e.g., Botvinick, 2008; Lashley, 1951; Zacks & Swallow, 2007; Zacks & Tversky, 2001), meaning that complex actions can be segmented into simpler actions. For example, preparing a burger consists of adding multiple ingredients, preparing the burger menu consists of preparing a burger, fries, and a drink, and fulfilling the order involves preparing, billing, and passing out the food. Although in everyday action control, we do not consciously name every simple action that makes up a more complex action, we are still able to segment them (i.e., to perceive them individually) if we need to (Newtson et al., 1977; for a review, see Zacks & Tversky, 2001). This segmentation of complex actions into simpler ones, or conversely, the combination of simpler actions into bigger events, is substantial for action control, as it makes it easier for our cognitive system to anticipate future actions (Kurby & Zacks, 2008; Lashley, 1951).

One growing area of the literature examines the control of simple actions and their interrelations via the investigation of feature integration and retrieval (see Frings et al., 2020). Here, simple actions like adding a slice of tomato to a burger are defined as short-term events, where stimulus, response, and effect features are integrated into a common representational format, so-called event files (Hommel, 1998; Hommel et al., 2001). If one of the integrated features is repeated at a subsequent event, the other integrated features are retrieved, affecting the execution of the current action. This influence on performance has been termed “binding effects.” Importantly binding effects are the result of two basic processes: feature integration and retrieval. While both are necessary for binding effects to occur, they can be independently influenced by additional factors (Frings et al., 2020). Integration and retrieval do not only occur in simple actions but are also found in action sequences, where consecutive responses are integrated into the same event representation and can retrieve each other, resulting in so-called response–response binding effects (Moeller & Frings, 2019a, 2019b). Here, sequentially executed responses are integrated into one event representation. If one of the responses now repeats at a next event, the other responses are retrieved. If the next required response matches one of the retrieved ones, executing this next response is facilitated. If they do not match, executing a response is impaired. With that, response–response bindings do not only follow the idea of hierarchical action control that more complex actions can be segmented into simpler actions, but they also allow for a more detailed view of the interrelations of simple actions. Moreover, response–response bindings are not limited to contiguous responses, but can also occur between noncontiguous responses and are not reliant on the temporal order of responses (Moeller & Frings, 2019a, 2019c). In addition, response–response bindings seem to be quite robust over time, without significant decay 6 seconds after integration (Geißler et al., 2021; Moeller & Frings, 2021b). With these characteristics, the concept of response–response bindings may apply to a broad range of complex actions.

In the context of response–response binding, looking at the segmentation between events means looking at the relation between responses. While we know that responses do not need to be temporally contiguous to be bound, it is still largely unknown how the spatial relation between responses influences response–response binding. Going back to the example of preparing a burger, all burger toppings are likely to be spatially separated in their containers rather than in one big pot to avoid confusion, which makes the preparation process more efficient. Likewise, the action to reach for a slice of tomato or cucumber follows this spatial separation, which might also add to efficiency. A look at the literature seems to indicate that the spatial separation between responses can indeed affect how they are cognitively represented.

In a variation of the Stroop task, spatially separating responses by increasing the distance between the responding hands facilitated responding (Lakens et al., 2011). When participants had to categorize the ink colors of letter strings (either color words or neutral strings, like *XXXX*) by button press, while ignoring the word meaning, responding correctly despite incongruent stimuli (e.g., the color word *blue* in red ink) became easier with more separation of response keys (Lakens et al., 2011; Nett & Frings, 2014). Lakens et al. (2011) argue that spatially structuring responses facilitates categorizations and that such structures can indeed affect cognitive processes. In other words, more distance between responses helped keep their representations apart (but see Schäfer & Frings, 2021).

Spatial separation between hands can also be induced via the placement of a barrier. Adding a barrier between two hands that received concurrent stimulation, helped to separate the processing of the interfering stimulation (Wesslein et al., 2015). Interestingly, this was the case even if the barrier was transparent, indicating that it is rather the perceived separation between hands than an obstructed vision that produced the effect (Wesslein et al., 2015).

Together, past findings seem to indicate that spatial separation of responding hands can affect response representation. What effect such separation of response representations has on binding and retrieval between these individual responses (i.e., on the coordination of actions) is so far unclear. Yet one might get an idea from investigations on other binding effects that manipulated separation of the integrated and retrieving features. A study on S-R-binding effects (Laub et al., 2018) suggests that separation furthers binding effects: Increased separability between a target and a distractor stimulus had beneficial effects on distractor-based retrieval, resulting in stronger distractor-response binding effects. That is, a separated stimulus started the retrieval process more efficiently. This was likely because such a separated distractor was more salient and thus received more attention, which generally benefits distractor-based retrieval (Moeller & Frings, 2014). Transferring this to response–response binding, we would expect that an increased separability of responses and thus more separated response representations might make the individual responses more distinct. This, in turn, may facilitate retrieval of previously integrated responses, resulting in overall larger response–response binding effects when responses are perceived as spatially separated.

In the present study, we examine whether the spatial separation of two responses affects binding effects between these responses. In a response–response binding paradigm, participants responded twice in the prime and twice in the probe. Here it can be assumed that the prime responses are integrated, so that repetition of one of them as the first probe response retrieves the other. Importantly, the two prime responses (and also the two probe responses) were given with different hands. Hence, we were able to measure integration and retrieval between two responses that were executed by different hands. We either placed a barrier between the hands giving these two responses, to induce separation, or did not separate the hands. In each prime and in each probe, participants gave responses (one with the left and one with the right hand) to two consecutive stimuli. If a barrier was placed between the hands, we assumed the responses to be perceived as more clearly separate. We expected this to result in stronger response–response binding effects in the condition where hands were separated by a barrier. To anticipate results, we did find larger response–response binding effects with separated hands, indicating that hierarchical action control might benefit from the spatial separation between individual actions.

## Experiment

### Method

#### Participants

For the baseline response–response binding effect, effect sizes across former studies (computed as t/sqrt(n)) were at least *d* = 0.63 (Moeller & Frings, 2019b). With α = .05 and a power of 1 − β = .8, 22 participants would be necessary to find a binding effect of this size (Faul et al., 2007). Ninety-six students (76 women) from Trier University participated in the experiment (barrier condition: *n* = 50, no-barrier condition: *n* = 46). A power analysis with the program G*Power assuming α = .05 and a power of 1 − β = .8 suggests that this sample was sufficient to find a between-subjects conditions difference of at least *d* = 0.58 (Faul et al., 2007). The samples’ mean age was 22.9 years (*SD* = 3.3). Five additional participants had to be excluded: One participant due to a high number of incorrect trials (181 out of 192), one due to outlier RTs (mean RTs of more than three times the interquartile range), and one due to outlier RTs and error rates (more than 20% errors, more than three times the interquartile range in both RTs and errors). Two participants used incorrect response keys (they did not use the number pad). All participants reported normal or corrected-to-normal vision and were rewarded with partial course credit or monetary compensation. The first part of the data was collected in a period from April 2021 to January 2022, with a brief recruitment stop during September 2021 due to the COVID-19 pandemic. After collecting data of 47 participants, the response–response binding effect in the control condition (no barrier, *n* = 22) was not significant, *t*(21)= 1.75, *p* = .096, *d* = 0.37. We then ran 49 more participants in the experiment, collecting data from another 24 participants in the control condition. For the full sample in the control condition, the binding effect was significant, *t*(45) = 4.70, *p* < .001, *d* = 0.69, *p*_augmented_ = [.0501, .0502] (Sagarin et al., 2014). The second part of the sample was collected from December 2022 to January 2023.^1^

#### Design

The design comprised two within-subjects factors—namely, Response A relation (response repetition vs. response change from prime to probe) and Response B relation (response repetition vs. response change from prime to probe), and one between-subjects factor—namely, barrier (barrier vs. no barrier).

#### Analysis

Data processing and analysis were done in R (R Core Team, 2019; Version 4.3.1). We compared the experimental conditions using a mixed analysis of variance (ANOVA) with Type III sums of squares. Additionally, we calculated the response–response binding effects as the advantage of probe Response A repetition (vs. probe Response A change) in probe Response B repetition trials minus the advantage of probe Response A repetition (vs. probe Response A change) in probe Response B change trials ([AcBr − ArBr] - [AcBc − ArBc]) as another way to represent the two-way interaction between Response A relation and Response B relation, that indicates binding between responses. Accordingly, this interaction can also be expressed as a *t* test against zero, with *F* = *t*^2^, whereas the *p* value for the interaction is equivalent to the *p* value for the t test.

#### Materials

The experiment was conducted using E-Prime 3.0. Instructions were presented in white on a black background on a standard liquid crystal display (TFT) screen. The viewing distance was approximately 60 cm. The list of possible stimuli consisted of eight different shapes with a height of 3.7° and a width of 4.0° of visual angle and made up of four overlapping lines of different lengths. The shapes could be presented in eight different colors (blue, green, red, yellow, purple, brown, and orange). In each display, two shapes were presented simultaneously 1.2° of visual angle to the left and right of the screen center. Participants responded via two out of four keys on a computer keyboard.

#### Procedure

Before the experiment, participants gave informed consent regarding the recording of personal data and responses during the experiment and indicated their age and gender. Instructions were given on the screen. Participants were instructed to place their middle and index fingers on the keys A, S, 5 (number pad), and 6 (number pad) of a standard computer keyboard. They were told that they would always see two line patterns that would be either identical or different in shape and identical or different in color. Their task was to first categorize the shapes (Response A) and then the colors (Response B) of these patterns as identical or different, by successively pressing two keys with the corresponding fingers. The left index and middle fingers were used for the shape classification. For identical shapes, participants were instructed to press the key with the left index finger (S) and for different shapes, they were supposed to press the key with the left middle finger (A). To classify the colors, the index and middle fingers of the right hand were used, respectively. For identical colors, a key was pressed with the right index finger (5), and for different colors, a key was pressed with the right middle finger (6).

**Fig. 1:**
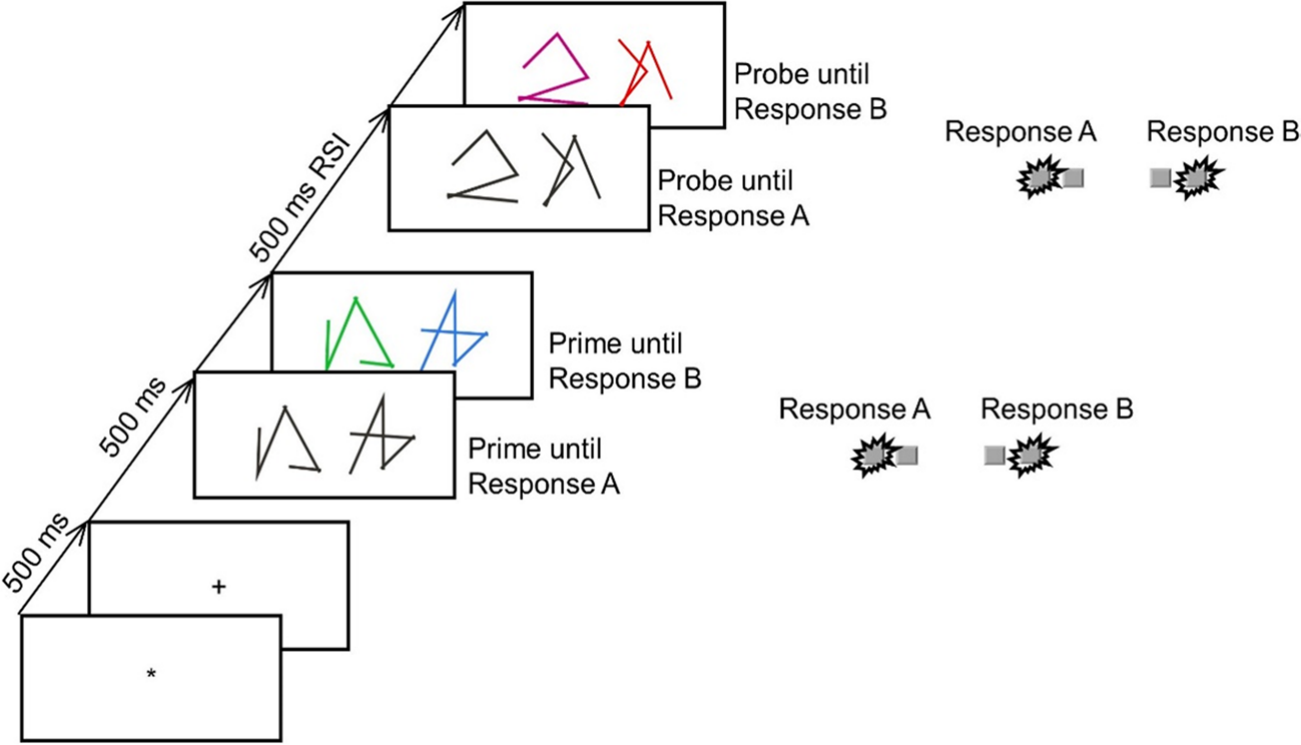
Sequence of events in one example trial. Note. Participants decided for each prime and each probe whether the presented stimuli had identical or different shapes (Response A) and identical or different colors (Response B). This is an example of a Response A repetition and Response B repetition trial. The stimuli are not drawn to scale; black is depicted as white and white as black. (Color figure online)

**Fig. 2:**
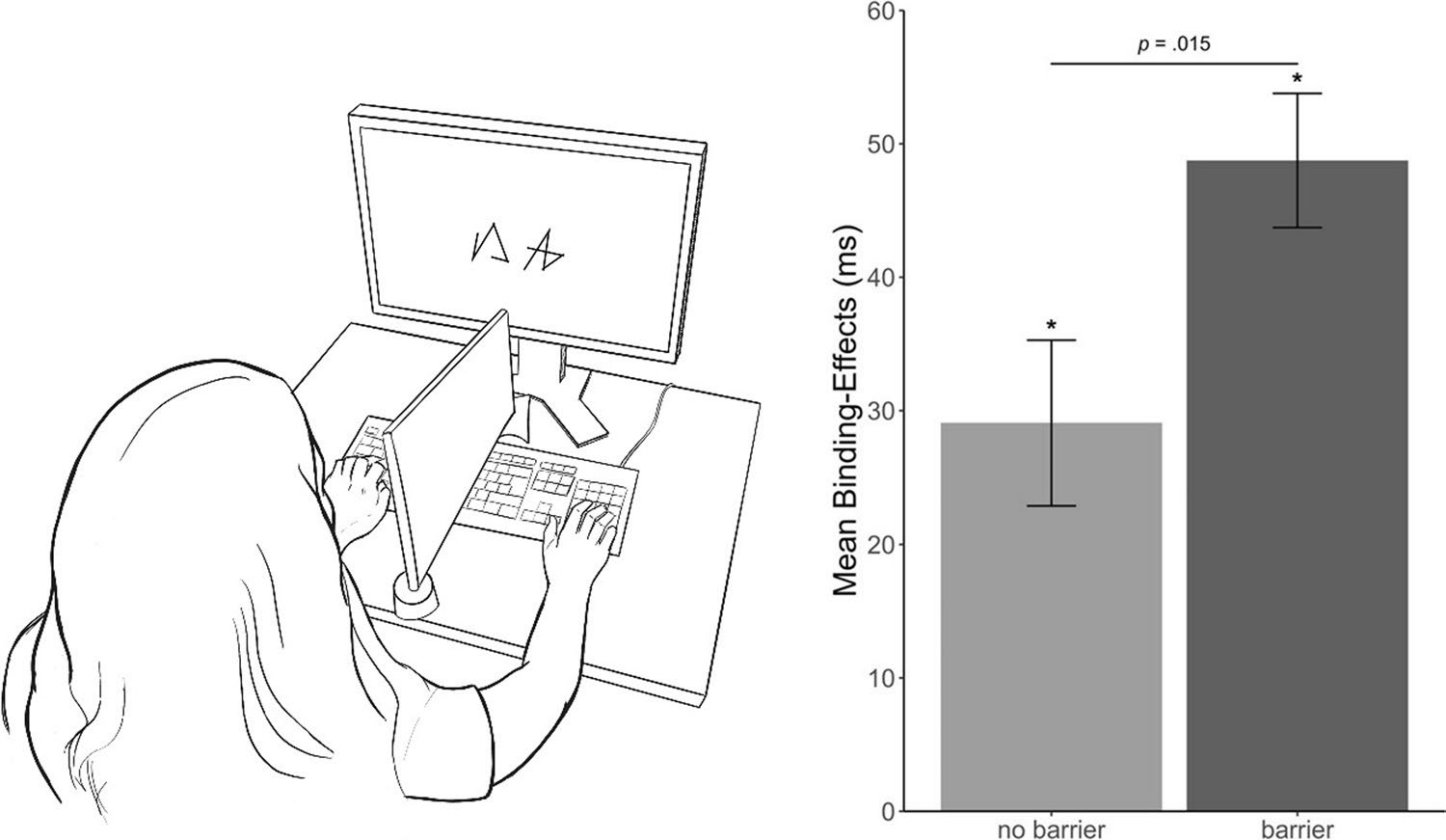
Visualization of the experimental set-up and results. Note. *Left:* Schematic depiction of the experimental setup. The participants’ hands were placed on the response keys at the respective ends of the keyboard. In the barrier condition, a black and opaque barrier was placed in the middle of the keyboard, lining up with the screen center, visually separating both halves of the keyboard but not the view of the screen. *Right:* Mean response–response binding effects for response times as a function of barrier (no barrier vs. barrier). Error bars depict the standard error of the mean. Binding effects are calculated as the advantage of probe Response A repetition (vs. probe Response A change) in probe Response B repetition trials minus the advantage of probe Response A repetition (vs. probe Response A change) in probe Response B change trials: [AcBr − ArBr] − [AcBc − ArBc]. *p < .05 indicates that binding effects differ significantly from zero

An asterisk that was presented for 500 ms in the middle of the screen indicated the beginning of each trial (see Fig. 1). Then a plus sign appeared for 500 ms, followed by the prime line patterns. These were presented in white for the shape comparison and, in the case of a correct response, changed color upon Response A execution (via the left hand). The colored shapes remained on the screen until Response B (via the right hand) was given. During training trials, a feedback message appeared on screen for 600 ms immediately following the response, indicating whether the given response was correct or not. Afterward, a blank screen appeared for 500 ms and was followed by the probe line patterns. The procedure in the probe was identical to that in the prime. Every 40 trials participants were allowed to take a short break, after which they resumed the task in their own time. In Response A repetition trials (Ar), the same response was required to the shapes of the prime and probe line patterns (e.g., the prime shapes differed, and the probe shapes differed). In Response A change trials (Ac), different responses were required for the categorization of the prime and probe line patterns (e.g., the prime shapes were identical, and the probe shapes differed). In Response B repetition trials (Br), the same response was required to the colors of the prime and probe line patterns (e.g., the prime colors were identical, and the probe colors were also identical). In Response B change trials (Bc), different responses were required to the prime and probe colors (e.g., the prime colors differed, and the probe colors were identical). These relations resulted in the four conditions Response A repetition with Response B repetition (ArBr), Response A repetition with Response B change (ArBc), Response A change with Response B repetition (AcBr), and Response A change with Response B change (AcBc). Each of these conditions was presented 12 times with each of the four possible combinations of identical/different shapes and colors in the probe, resulting in 192 experimental trials total. Shapes and colors were randomly assigned to the different positions/displays while restricting that neither could repeat between prime and probe of one trial. In the beginning, participants practiced their task for 16 trials (subsample of the experimental trials). Half of the sample completed the experiment with a barrier separating both hands (see Fig. 2), placed there before the start of the experiment (barrier condition). The other half completed the experiment without separation by a barrier (no barrier condition).^2^

### Results

The dependent variable of interest was probe Response B performance. Regarding the analysis of response times (RTs), only trials with correct responses A and B in both prime and probe were considered. The error rate for prime responses (A or B) was 7.0%. The probe error rates were 2.8% for Response A and 4.4% for Response B (only including trials with correct previous responses). We excluded RTs of more than 1.5 interquartile ranges above the third quartile of the probe Response B RT distribution of the participant (Tukey, 1977) and RTs shorter than 200 ms from the analysis. Due to these constraints, 17.7% of the trials were excluded from the RT analyses. For the mean RTs and error rates, see Table 1. For RTs and error rates of prime Response B and probe Response A see the Appendix Tables 2 and 3.

**Table 1:** Mean response times (in milliseconds) and mean error rates (in percentages) for probe Response B, as a function of Response A relation between prime and probe, Response B relation and barrier

	No barrier		Barrier	
	*B repetition*	*B change*	*B repetition*	*B change*
*A change*	576 (6.9)	555 (2.7)	585 (5.4)	559 (2.0)
*A repetition*	552 (4.6)	561 (5.8)	553 (3.3)	575 (5.6)

In a 2 (Response A relation: repetition vs. change) × 2 (Response B relation: repetition vs. change) × 2 (barrier: no barrier vs. barrier) analysis of variance (ANOVA) on probe Response B RTs, the main effect for Response A relation was significant, *F*(1, 94) = 13.13, *p* < .001, η_p_^2^ = .12, while the main effects for Response B relation, *F*(1, 94) = 2.89, *p* = .092, η_p_^2^ = .03, and barrier, *F*(1, 94) = 0.19, *p* = .666, η_p_^2^ < .01, were not. The interaction of Response A and Response B relation was significant, *F*(1, 94) = 96.47, *p* < .001, η_p_^2^ = .51, indicating binding between responses. Importantly, this was further modulated by barrier, *F*(1, 94) = 6.16, *p* = .015, η_p_^2^ = .06. Follow up analyses revealed a larger binding effect in the barrier condition, *t*(49) = 9.69, *p* < .001, *d* = 1.37, than in the no barrier condition, *t*(45) = 4.70, *p* < .001, *d* = 0.69. For a summary of mean binding effects, see Fig. 2.

The same analysis on error rates revealed no significant main effects for Response A relation, *F*(1, 94) = 3.15, *p* = .079, η_p_^2^ = .03, and barrier, *F*(1, 94) = 2.44, *p* = .121, η_p_^2^ = .03, but for Response B relation, *F*(1, 94) = 6.96, *p* = .010, η_p_^2^ = .07. Again, the interaction of Response A and Response B relation was significant, *F*(1, 94) = 53.88, *p* < .001, η_p_^2^ = .36, with significant binding effects in the barrier condition, *t*(49) = 6.63, *p* < .001, *d* = 0.93, as well as in the no barrier condition, *t*(45) = 4.23, *p* < .001, *d* = 0.62. However, this relation was not further modulated by barrier, *F*(1, 94) = 0.07, *p* = .787, η_p_^2^ < .01.

### Discussion

In the present study, we investigated the influence of hand separation on response–response binding effects. Every trial consisted of a prime and a probe, each with two consecutive Responses A and B that were given with alternating hands. Replicating earlier studies (e.g., Moeller & Frings, 2019b; Selimi et al., 2022), we found a significant response–response binding effect. Importantly, this effect was modulated by the presence/absence of a barrier between the hands. Separation of the hands through the placement of a barrier led to significantly larger binding effects than without a barrier. Binding effects can be interpreted to indicate what becomes part of a common action representation (Hommel, 2009; Moeller & Frings, 2019b). Apparently, the placement of a barrier between the spatial positions of individual responses influences the degree to which these responses, when integrated into a higher-order representation, can then modify subsequent actions.

Binding effects are the result of two processes, namely integration (during the prime) and retrieval (during the probe), working together (Frings et al., 2020). In our study, the manipulation of response separation might have affected either response integration, response retrieval, or both processes. Even though we cannot pinpoint the exact process with this study, it is reasonable to assume that it was the retrieval process that was affected by the separation manipulation. For one, there is growing evidence that retrieval is more easily and more often influenced by modulations than integration (Hommel, 2022; Hommel et al., 2014; Moeller & Frings, 2014). In addition, the same pattern of beneficial effects due to separability of retrieving features was reported in the past: While feature integration is largely unaffected by separation, the reencounter of an easily separable feature facilitated retrieval compared with a less separable feature (Laub et al., 2018). Even though this was not the focus of the current study, to get definite evidence as to whether integration or retrieval was affected in the present study, the separation manipulation would have to be applied to the prime (associated with integration) and probe (associated with retrieval) separately (see Laub et al., 2018; Schmalbrock et al., 2022). Unfortunately, such a manipulation in the currently used paradigm is impossible to conduct, as it would involve a change of setup every two responses. Yet a conceptually similar question might be interesting to investigate in a virtual reality setup.

Evidence for response–response binding effects can be interpreted as an indication that two individual responses are represented in one higher-order representation (Moeller & Frings, 2019b). From this angle, our results suggest that the separation of responding hands affects the representation of such response sequences, with stronger response–response binding with increased separability. An explanation might be that separation helps to structure events cognitively: Although an overarching event is formed around the two responses, it is reasonable to assume that these responses also include smaller event representations (i.e., event files containing a single response; Moeller & Frings, 2019b, 2021a). Thus, binding effects seem to have a hierarchical structure, but importantly, individual parts of larger-scale events still seem to retain their individual representation to some degree. The separation of responses induced by the barrier apparently helped to cognitively separate responses and could have thus led to more distinct individual representations of each response. Such more distinct cognitive representations of individual responses might then facilitate piecing them together into an action sequence.

The beneficial effects of distinct representations of individual actions seem to be in line with other research on hierarchical actions. Findings in the event segmentation literature indicate that we naturally segment actions of different complexity in time (i.e., we set temporal boundaries between events. This happens on the level of complex everyday actions (e.g., Newtson et al., 1977; Zacks, Braver, et al., 2001a; Zacks, Tversky, & Iyer, 2001b), but also on the level of individual or few responses (Fournier & Gallimore, 2013). Boundaries seem to be similar across different participants (e.g., Zacks, Tversky, & Iyer, 2001b) and individuals who are better at segmenting larger-scale events, are also better at remembering them later on (Zacks et al., 2006). Thus, even if it is not specifically instructed, our cognitive system uses segmentation to make sense of the world and predict future actions (Kurby & Zacks, 2008; Lashley, 1951). In our study, the spatial separation of individual actions by a barrier probably induced more distinct representations of individual actions and might have thus supported the natural tendency to segment events.

Notably, the present design included a task switch in each prime and each probe response pair: participants first indicated whether shapes were identical or not and second, whether colors of the shapes were identical or not. That is, repetition of the task (e.g., from Prime Response B to Probe Response B) might have caused retrieval of bound features from the first of these responses (Kübler et al., 2022; Pösse et al., 2006; Waszak et al., 2003) and the repetition of task pairs (A and B) might have facilitated responding in general (Hirsch et al., 2017, 2018). However, since task (pair) repetitions occurred in every trial in the present study, neither effect can account for the observed differences in the result pattern. In addition, both tasks (even though responded to with different hands) included the decision whether identical or different stimulus features (shapes or colors respectively) were presented on the screen. Response–response correspondence effects have been known to affect performance depending on task switching (Lien et al., 2003) with a decrease in performance of corresponding responses if the task switches (Koch et al., 2011; Schuch & Koch, 2004). Indeed, prime Responses B were significantly longer with compatible than with incompatible prime responses, in the present study (602 vs. 591 ms), *t*(95) = 2.32, *p* = .023, *d* = 0.12. This was not the case for probe responses (562 vs. 568 ms), *t*(95) = −1.69, *p* = .095, *d* = −0.07. Importantly, this effect within primes and probes cannot account for our main finding that RR binding effects are larger for separated than for nonseparated responses.

To sum up, with the present study we analyzed an important factor affecting mechanisms in the coordination of action sequences. Apparently, short-term integrations between individual responses in an action sequence affect further actions more, with increasing distinction between the responses. This may indicate that the control of complex actions is more efficient with clear distinctions of individual lower-level actions.

## Data Availability

The datasets and analysis code of the current study are available in the Psycharchives repository under 10.23668/psycharchives.7961 (dataset) and 10.23668/psycharchives.7960 (analysis code). The experiment was not preregistered.

## Appendix

**Table 2:** Mean response times (in milliseconds) and mean error rates (in percentages) for probe Response A, as a function of Response A relation between prime and probe, Response B relation and barrier

	No barrier		Barrier	
	*B repetition*	*B change*	*B repetition*	*B change*
*A change*	830 (3.0)	837 (4.0)	813 (2.2)	818 (3.1)
*A repetition*	832 (3.4)	836 (3.1)	826 (2.1)	813 (2.2)

**Table 3:** Mean response times (in milliseconds) and mean error rates (in percentages) for prime Response B, as a function of barrier.

No barrier	Barrier
595 (4.6)	598 (4.2)

## Footnotes

1 To account for the two data collection times also in an analysis of the entire sample, we conducted a control analysis including the factor “time of measurement.” In a 2 (Response A) × 2 (Response B) × 2 (barrier) × 2 (time of measurement) ANOVA on probe Response B RTs, the factor time point did not significantly modulate response–response binding effects, *F*(1, 92) = 1.71, *p* = .195, η_p_^2^ = .02, or the modulation of binding effects by barrier, *F*(1, 92) = 3.03, *p* = .085, η_p_^2^ = .03. In the same analysis on error rates, again neither the binding effect, *F*(1, 92) < 1, *p* = .676, η_p_^2^ < .01, nor its modulation by barrier, *F*(1, 92) < 1, *p* = .709, η_p_^2^ < .01, were further modulated by time of measurement. Any descriptive interaction with time point of measurement was of the ordinal type—that is, the pattern was identical in both time points of measurement (RR binding effects with barrier > RR binding effects without barrier).

2 Some of these data were collected as part of a larger experiment. There were other conditions with different participants that are not relevant to the question of the current study.
